# Multiple pollutants in groundwater near an abandoned Chinese fluorine chemical park: concentrations, correlations and health risk assessments

**DOI:** 10.1038/s41598-022-07201-8

**Published:** 2022-03-01

**Authors:** Jiaxi Tang, Yongle Zhu, Biao Xiang, Yu Li, Ting Tan, Ying Xu, Mengxue Li

**Affiliations:** 1 College of Environmental Science and Engineering, Liaoning Technical University, Fuxin, 123000 China; 2 Liaoning Academy of Agricultural Sciences, Shenyang, 110161 China

**Keywords:** Environmental chemistry, Environmental impact, Public health

## Abstract

Contamination and adverse effects from various pollutants often appear in abandoned industrial regions. Thus, nine groundwater samples were collected from the vicinity of the fluorochemical industry in Fuxin City, Liaoning Province, to determine concentrations of the ten heavy metals arsenic (As), chromium (Cr), cadmium (Cd), lead (Pb), nickel (Ni), copper (Cu), manganese (Mn), zinc (Zn), iron (Fe) and mercury(Hg), as well as those of fluorine (F^−^) and eighteen poly- and perfluorinated substances (PFASs), analyse correlation relationships, and assess the health risks for different age groups. The results showed that the levels of fluorine (F^−^) (0.92–4.42 mg·L^−1^), Mn (0.0005–4.91 mg·L^−1^) and Fe (1.45–5.61 mg·L^−1^) exceeded the standard limits for drinking water. Short chain perfluorobutanoic acid (PFBA) (4.14–2501.42 ng·L^−1^), perfluorobutane sulfonate (PFBS) (17.07–51,818.61 ng·L^−1^) and perfluorohexanoic acid (PFHxA) (0.47–936.32 ng·L^−1^) were the predominant substances from the PFASs group. No individual PFASs levels had significant relationships with F^−^ or heavy metal contents. There was a positive relationship between short chain PFASs concentrations and water depth and a negative relationship between long chain PFASs concentration and water depth. The hazard quotient (HQ) decreased in the order F^−^ > heavy metals > PFASs and also decreased for older age groups. In addition, As, Fe, Mn and perfluorooctanoic acid (PFOA) were the main sources of risk from the heavy metal and PFASs groups, respectively.

## Introduction

Multiple pollutants exist in environmental media and are widely distributed in areas surrounding sites of intensive industrial and agricultural activities. These pollutants have adverse effects on the ecological environment and human health and exhibit complex behaviour that differs from the existing condition. Many studies have demonstrated that processes involving serious contamination by different pollutants tend to be more complicated than those involving single pollutants^1–3^. Although some persistent organic pollutants (POPs) have been banned from use in packaging, smelting, paper making, pesticides, and so on, dangerously high concentrations have always been detected in different environmental media^4–6^.

Heavy metals are the most common pollutants distributed in areas surrounding industrial sites^7,8^. Moreover, some emerging organic pollutants also merit greater consideration. Poly- and per-fluorinated substances (PFASs) are typical pollutants released by the fluorine chemical industry and are a class of anthropogenic organofluorine compounds in which each hydrogen atom on an alkyl chain is replaced by a fluorine atom^9,10^. They have unique physicochemical characteristics arising because there are more fluorine atoms than H atoms, and the strengths of carbon–fluorine bonds give fluoroalkyl moieties high thermal, chemical and biochemical stability. Whether from heavy metals or PFASs, harmful effects have negative impacts on systems in the human body, including respiratory, urinary, immune, and even reproductive systems^11–14^. As indicated in previous studies, complexes formed by self-assembly through bidentate or multidentate coordination of organic ligands and heavy metals change the existing forms of pollutants^2^. In addition, the toxicities of pollutants are dependent on their existing forms, and transition pollutants change their solubility and bioavailability via complexation^2,3^.

A special feature in our study region should also be taken into consideration: a high F background level exists in local surface and groundwater. High-F groundwater has adverse effects on the human body if residents use contaminated water as a drinking source for long periods. Additionally, Li et al. (2001) examined 8266 male and female subjects > 50 years old as a study group and demonstrated that the overall risk of fractures, as well as those for hip fractures in particular, increase when the F^−^ concentration in drinking water exceeds 4.32 mg·L^−1^^15^. A previous study of heavy metal contamination in an agricultural area of Fuxin City found that the mean concentrations of As, Cd, Cr, Ni and Zn, but not those of Cu, Hg, and Pb, exceeded their respective background values^16^. Importantly, Ni, Cr, and As were the main carcinogenic pollutants, and ingestion and dermal contact were the major exposure pathways; these metals posed potential health risks to adults and children^16^. High concentrations of heavy metal and PFASs contaminants were reported for soils located near fluorine chemical park (FCP) in our previous studies and other studies^16–18^. The PFASs concentration reached μg/L levels in surface water, which indicated that Fuxin City has been severely impacted by industrial activities at the FCP^18^.

Local residents have always used contaminated groundwater as a source of drinking water; however, the health risk from the groundwater exposure pathway may be higher than that in soil^19^. Thus, we improved the monitoring of heavy metals and considered the high background level of F in groundwater. Then, PFASs, which are typical pollutants in FCP, were added to the pollutant list. While correlation relationships among a variety of pollutants are always limited, different pollutant assessments in groundwater systems should also be carried out. Thus, the objective of our study is (i) to identify the concentrations of the heavy metals arsenic (As), chromium (Cr), cadmium (Cd), lead (Pb), nickel (Ni), copper (Cu), manganese (Mn), zinc (Zn), iron (Fe) and mercury(Hg) and those of inorganic F^−^ substances, ammonia nitrogen (NH^+^_4_-N), nitrite nitrogen (NO^−^_2_-N), nitrate nitrogen (NO^−^_3_-N) and PFASs; (ii) to determine the correlations between various pollutants; and (iii) to evaluate the health risks from heavy metals, F^−^ and PFASs for different age groups. Our work will provide scientific guidance and helpful support for implementation of pollutant control measures by local policy-makers.

## Methods and materials

### Study area

Our study area, Fuxin City of Liaoning Province, is one of the most important fluorine chemical center in China. The FCP is close to the Xi River and covers a total area of 7.1 square kilometers; the central coordinates are 121° 35ʹ E and 41° 56ʹ N. Industry in the park mainly focuses on fluorine compounds, the development of inorganic fluorides, aliphatic fluorides, fluorine-containing polymer materials and other core industrial projects. The main products are p-aminotrifluoromethoxybenzene, electrolytic fluorine series products, sodium tetrafluorpropionate, fluorocarbon alcohol, tetrafluoro coatings, etc. In 2013, the local government decided to move several fluorine chemical enterprises and expand their scale to accelerate economic development of the fluorine chemical industry.

### Sample collection and pretreatment

**Figure 1:**
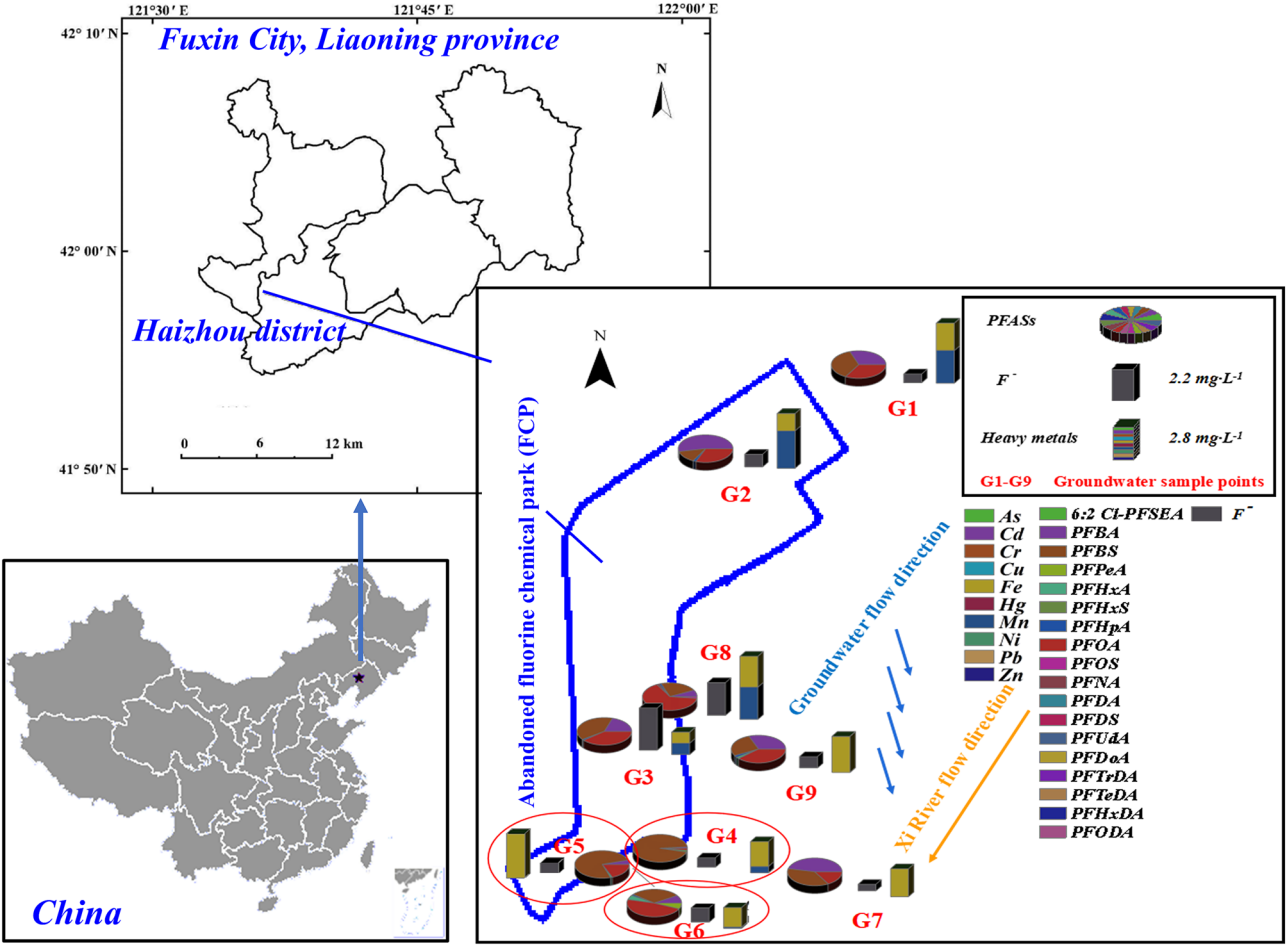
Groundwater sampling points and pollutant concentrations from the Fluorine Chemical Park (FCP) of Fuxin City, Liaoning Province.

The study area and sampling points are listed in Fig. 1, and Google Earth Pro (V7.3.3.7721) software was applied to establish the latitudes and longitudes of each sampling point. Groundwater samples (sites G1–G9, n = 9) were collected from civic wells during August and September 2019. In the sampling process, each polyethylene bottle was rinsed 1–2 times with pure water and 2–3 times with the groundwater sample before sampling. Groundwater samples were collected at 3–5 m below the ground for storage in two cleaned 1 L PP bottles. One was used for heavy metal determination, and one was used for determining physicochemical properties and F^−^ and PFASs levels. Then, 2 mL of 1% HNO_3_ was added to each water sample for heavy metal determination to stabilize the forms of heavy metals. A pH < 2 was established with HNO_3_ before storage at 4 °C.

The physicochemical properties of the water samples were determined in the laboratory. Ten heavy metals, As, Cr, Cd, Pb, Ni, Cu, Mn, Zn, Fe, Hg and F^−^ were analysed as target pollutants in groundwater, and all experimental methods for determining physicochemical properties, heavy metals and F^−^ from precise standards are given in Supplementary Table S1 online. All standard values for heavy metals and F^−^ in drinking water are from GB/T14848-2017 (Class III)^20^ and were compared with the pollutant concentrations determined in the present study.

Next, PFASs (abbreviation for Supplementary Table S2 online) pretreatment methods were developed by Chen et al.^18^ and Bao et al.^21^. Oasis WAX cartridges (500 mg, 6 mL, Waters, USA) were used to extract PFASs from the groundwater samples. The WAX SPE cartridges were conditioned with 5 mL of methanol containing 0.1% ammonium hydroxide, 5 mL of methanol, and 5 mL of Milli-Q water. The water samples were then loaded onto the cartridges at a rate of 1 drop/s. The cartridges were washed with 5 mL of 2.5 mM acetic acid/ammonium acetate (pH = 4) and then centrifuged for 10 min at 3000 rpm to remove residual water. Ionizable compounds were eluted with 5 mL of methanol and 5 mL of methanol containing 0.1% ammonium hydroxide. The eluent was concentrated under nitrogen and dissolved in 1 mL of acetonitrile. Then, the liquid was filtered by 0.45 μm membrane and stored in 1.5L vials for machine detection and analysis. All PFASs concentrations were analysed via high-performance liquid chromatography triple quadrupole mass spectrometry (HPLC–MS/MS), and measurement parameters are listed in Supplementary Tables S3 and S4 online.

### Health risk assessment

Noncarcinogenic methods were applied to assess potential health risks^22^. The health risks of F^−^, heavy metals, and PFASs in groundwater were assessed for the different age groups 6–12 months, 6–11 years, 11–16 years, 16–18 years, 18–21 years, 21–65 years and > 65 years. Although local residents have various ways of contacting these pollutants, direct ingestion of a drinking water source is always thought to be the most harmful exposure method. The hazard quotient (HQ) is typically used in noncarcinogenic assessments, and the mixed hazard quotient (HQ_mix_) is based on the sum of HQ values for single pollutants. If the HQ/HQ_mix_ for an assessed pollutant exceeds 1, risks to human health are indicated. If the HQ/HQ_mix_ for the assessed pollutant is below 1, the risk is within the acceptable range. The formulas for HQ/HQ_mix_ calculation are given in Eqs. (1) to (3):

1 $$DWEL = \frac{ADI \times BW}{{DWI \times AB \times FOE}}$$

2 $$HQ = \frac{C}{DWEL}$$

3 $$HQ_{mix} = \sum\limits_{i = 1}^{n} {HQ = \sum\limits_{i = 1}^{n} \frac{C}{DWEL} }$$

where ADI (μg/kg day^−1^) is the acceptable daily intake without significant health risk, BW (kg) represents percentile body weight, DWI (L day^−1^) is the drinking water intake in daily life, AB defines the gastrointestinal absorption rate (assumed to be equal to 1), FOE is the frequency of exposure (350 days/365 days = 0.96) and n in Eq. (3) is used to assess the potential risks for different pollutants. These parameter values can be found in Supplementary Table S5 online.

### QA/QC

To determine the accuracy of all pollutant analyses, all pretreatment and detection methods for heavy metals, F^−^ and PFASs were taken from standard reference materials or prior studies^23–25^. Three analytical duplicates were included in the analysis of each water sample. A field blank was used to eliminate the influence from the sampling bottle on concentration, and a series of laboratory Milli-Q water samples was added into the sampling bottle to determine this free blank. Procedural blanks were identified for every batch of samples, and they were either not detected (nd) or lower than the limits of quantification (LOQs) to ensure data accuracy and method availability. LOQs were defined as the minimum injection volume needed for reproducible measurements of peak area within ± 20% for repeated injections. All water samples were membrane treated to eliminate impurities. The limits of detection (LODs) and LOQs for F^−^ and heavy metals are shown in Supplementary Table S1 online. The recovery rates for heavy metals ranged from 88.42–97.49%, and the relative standard deviations (RSD) ranged from 3.55 to 6.69%. The PFASs concentrations were identified with a concentration series containing 0, 0.01, 0.1, 1, 5, 10, 50, 100, and 500 μg·L^−1^ used to draw the standard curve (R^2^ > 0.99). An internal solution (IS, see Supplementary information) was added to each sample to assess the accuracy for recovery of analytes, and acceptable recovery levels for 11 IS substances ranged from 98% ± 5% to 119 ± 2%. The LODs and LOQs of PFASs were applied in Supplementary Table S3 online.

### Standard analyses

Statistical analysis and significance tests were analysed with IBM SPSS statistics 22 (USA). Origin 2021 and ArcGIS 10.7 were used to make tables and figures.

## Results and discussion

### Characteristics of various pollutants in water

#### NO^−^_2_-N, NO^−^_3_-N, NH^+^_4_-N and F^-^

Pollutant concentrations in groundwater from the FCP of Fuxin City are shown in Table 1. The NO^−^_2_-N and NO^−^_3_-N concentrations ranged from no detection (nd) to 0.063 mg·L^−1^ and nd to 10.77 mg·L^−1^, which were below the standard values of 1.00 mg·L^−1^ and 20.00 mg·L^−1^, respectively (Table 1). The mean NH^+^_4_-N concentration (1.02 mg·L^−1^) was two times that of the standard value (0.5 mg·L^−1^) in GB/T14848-2017^20^. Three major sources of NH ^+^_4_-N were identified: leather industry production, introduction of nitrogen chemicals, and human activities^26^.

**Table 1:** Concentrations of various pollutants in ground water (mg·L^−1^) from the FCP of Fuxin, China.

Indicators	G1	G2	G3	G4	G5	G6	G7	G8	G9	Standard value^a^
NO^−^_2_-N	0.063	0.004	Nd	0.016	0.028	0.049	0.007	0.002	0.005	≤ 1.00
NH^+^_3_-N	0.77	4.68	0.60	0.23	0.14	2.04	0.18	0.52	0.02	≤ 0.50
NO^−^_3_-N	6.13	Nd	Nd	5.33	9.02	0.48	10.90	0.14	10.77	≤ 20.00
F^−^	0.92	1.32	4.42	0.99	1.07	1.50	0.69	3.43	1.18	≤ 1.0
SO_4_^−^	355	196	6.84	652	217	274	504	442	521	≤ 250
Cl^−^	209	89.3	255	191	219	201	85.9	258	276	≤ 250
As	0.0038	0.01	0.0049	0.0045	0.01	0.01	0.0025	0.01	0.01	≤ 0.01
Cd	Nd	Nd	Nd	Nd	Nd	Nd	Nd	Nd	Nd	≤ 0.005
Cr	0.0030	0.0014	0.0027	0.0005	0.0031	0.0005	Nd	0.0027	0.0021	≤ 0.05
Cu	0.0008	Nd	0.01	0.0049	0.0013	0.0014	Nd	Nd	0.01	≤ 1.00
Fe	3.60	2.28	1.45	3.28	5.61	2.57	3.68	4.07	4.72	≤ 0.3
Hg	Nd	Nd	Nd	Nd	Nd	Nd	Nd	Nd	Nd	≤ 0.001
Mn	4.29	4.91	1.49	0.83	0.01	0.16	0.0005	4.22	0.004	≤ 0.1
Ni	0.0034	Nd	Nd	0.0041	0.01	0.0007	0.0013	0.0033	0.01	≤ 0.02
Pb	Nd	Nd	0.0003	0.0004	Nd	Nd	Nd	Nd	Nd	≤ 0.01
Zn	Nd	Nd	Nd	Nd	Nd	0.0020	Nd	Nd	Nd	≤ 1.00

^a^Standard value from GB/T14848-2017 (Class III).

*Nd* not detected.

A high F^−^ background always exists in the local groundwater. Abundant fluorite (CaF_2_) resources can dissolve and disperse along the water flow direction, which was identified as the main source of F^−^ in groundwater. The highest F concentration appeared in G3 (4.42 mg·L^−1^), followed by G8 (3.43 mg·L^−1^) and G6 (1.50 mg·L^−1^) (Table 1 and Fig. 1). These concentrations far exceeded the limit (1.0 mg·L^−1^) for Class III Chinese drinking water, although F concentration standards from different organizations and institutes are always controversial^20^. The U.S. Environmental Protection Agency (EPA) recommends fluoridated community water systems adjust fluoride to approximately 0.7 mg·L^−1^^27^. Additionally, other studies have indicated that only F^−^ concentrations below 2 mg·L^−1^ preclude adverse effects on the human body from drinking water^28^. Dissanayake et al. suggested that if the F concentration in drinking water exceeds 1.5 mg·L^−1^, the probability of dental fluorosis increases for children^29^. Regardless of which standard values are used, all F values in our study exceeded the screening or risk values, which indicated that F contamination in groundwater may pose a threat to the human body.

#### Heavy metals

As, Cr, Cu, Fe, Mn, Ni, Pb, and Zn were all detected, whereas Cd and Hg were assigned nd status. The predominant heavy metals were Mn (0.0005–4.91 mg·L^−1^), Fe (1.45–5.61 mg·L^−1^) and As (0.0025–0.01 mg·L^−1^) (Table 1 and Fig. 1). In our previous studies, the levels of Ni (63.01 mg·kg^−1^), Cr (48.26 mg·kg^−1^), Cu (23.29 mg·kg^−1^), Pb (12.54 mg·kg^−1^) and As (9.80 mg·kg^−1^) in soil were higher than those in groundwater^19^. Surprisingly, Cd and Hg were detected in soil but not in groundwater. Heavy metals entering the soil have weak transport capacity and are easily adsorbed by soil colloids; moreover, the physical and chemical properties of soil affect the form adopted, especially for some metals that have difficulty migrating from soil to water^2,12^. The concentrations of the two heavy metals Fe and Mn were much higher than 0.3 mg·L^−1^ and 0.1 mg·L^−1^, especially for Fe, which exceeded the standard value at all sampling points. Rich ore resources are the main sources of excess Fe and Mn in groundwater, and metal powder can enter groundwater during the extraction process.

#### PFASs

Eighteen PFASs groundwater concentrations from the FCP of Fuxin City are shown in Table 2. The ∑_18_PFAS concentrations ranged from 72.49 ng·L^−1^ to 68,142.14 ng·L^−1^, and the mean value was 11,108.42 ng·L^−1^. Short chain PFBA (4.14–2501.42 ng·L^−1^), PFBS (17.07–51,818.61 ng·L^−1^) and PFHxA (0.47–936.32 ng·L^−1^) were the predominant substances (Table 2 and Fig. 1). The highest ∑_18_PFAS concentrations were found at G5 (68,142.16 ng·L^−1^), G4 (26,464.56 ng·L^−1^) and G6 (3334.00 ng·L^−1^), which were very near the center of the FCP (Fig. 1 and Table 2). Short chain PFASs, as alternatives to long chain substrates, have been widely used in the FCP of Fuxin City or in other countries^21^. The PFASs contamination in our study area was much higher than those in other regions, including Taiwan^30^, the Maozhou River basin^28^, a Tianjin suburb^31^ and Hubei Province^32^. Short chain PFASs (PFBA, PFBS, PFHpA, PFHxA and PFHxS) and PFOA are commonly detected currently due to the heavy use of long chain substitutes and different industry types^17,32^. There is no current environmental standard for PFASs in China, whereas newly emerging pollutants have been of concern in Canada^33^, Swedish^13^, American^34^ and so on. The U.S. Environmental Protection Agency (USEPA) limit for PFOA in drinking water is 400 ng·L^−1^, and the PFOA concentration (35.34–11,305.65 ng·L^−1^) exceeds the standard value by several orders of magnitude^35^. The low concentration detected for long-chain PFASs (C ≥ 8) may result mainly from historical industry production and emissions. Short chain PFASs applied to improve hydrophilicity and fluidity tend to migrate easily in water bodies, whereas long chain PFASs are readily adsorbed by large particles or sediments^36^. The Xi River is the main river flowing through the FCP and receives many industrial pollutants throughout the year. Surface water recharge and infiltration moves PFASs into the groundwater system. Based on contamination in the Maozhou River basin, a potential link between the surface and groundwater was identified, and the PFASs concentration in surface water had a highly positive relationship with that in groundwater^37^. A PFOS substitute of 6:2 Cl-PFSEA was detected at low concentrations (0.01–0.17 ng·L^−1^), which indicates that the emerging PFASs substitute has been used in local industrial production^38,39^ (Table 2). In addition, PFSAs concentrations were much higher than PFCAs concentrations. Different functional groups cause PFASs to exhibit varied environmental behaviour, especially short chain PFASs, which migrate easily in water bodies^40^.

**Table 2:** PFASs concentrations in groundwater (ng·L^−1^) from the FCP of Fuxin, China.

PFASs	G1	G2	G3	G4	G5	G6	G7	G8	G9
6:2 Cl-PFSEA	0.17	0.11	0.03	0.01	0.01	0.03	0.01	0.40	0.02
PFBA	57.21	71.84	137.31	277.23	2501.42	234.20	115.56	4.14	204.79
PFBS	58.57	17.07	290.94	25,025.59	51,818.61	1140.46	93.40	18.36	145.46
PFPeA	0.37	1.24	2.46	34.04	313.35	170.90	0.58	0.70	6.12
PFHxA	1.12	0.90	5.06	128.63	936.32	139.96	1.20	0.47	11.20
PFHxS	nd	0.12	1.02	412.19	539.60	8.57	0.17	nd	0.17
PFHpA	1.47	2.92	7.82	98.29	681.07	43.39	1.61	1.10	10.90
PFOA	63.34	44.93	305.39	433.33	11,305.65	1581.99	35.34	44.54	253.67
PFOS	0.43	0.18	1.65	55.17	43.94	7.60	0.35	1.03	0.22
PFNA	1.83	0.10	0.45	Nd	1.88	5.29	0.07	1.26	0.10
PFDA	1.14	0.11	0.41	0.02	Nd	1.54	0.08	0.31	0.04
PFDS	0.05	Nd	Nd	Nd	Nd	Nd	Nd	Nd	Nd
PFUdA	0.54	0.02	0.04	Nd	Nd	Nd	Nd	0.06	Nd
PFDoA	0.62	0.04	Nd	Nd	Nd	0.03	Nd	0.02	0.02
PFTrDA	0.29	0.04	Nd	Nd	Nd	Nd	Nd	Nd	0.04
PFTeDA	0.53	0.04	Nd	Nd	Nd	Nd	Nd	0.02	0.03
PFHxDA	0.42	0.04	Nd	Nd	Nd	0.01	Nd	Nd	0.03
PFODA	0.75	0.08	0.06	0.06	0.32	0.04	Nd	0.06	0.09

*Nd* not detected.

### Correlation relationship analysis

**Figure 2:**
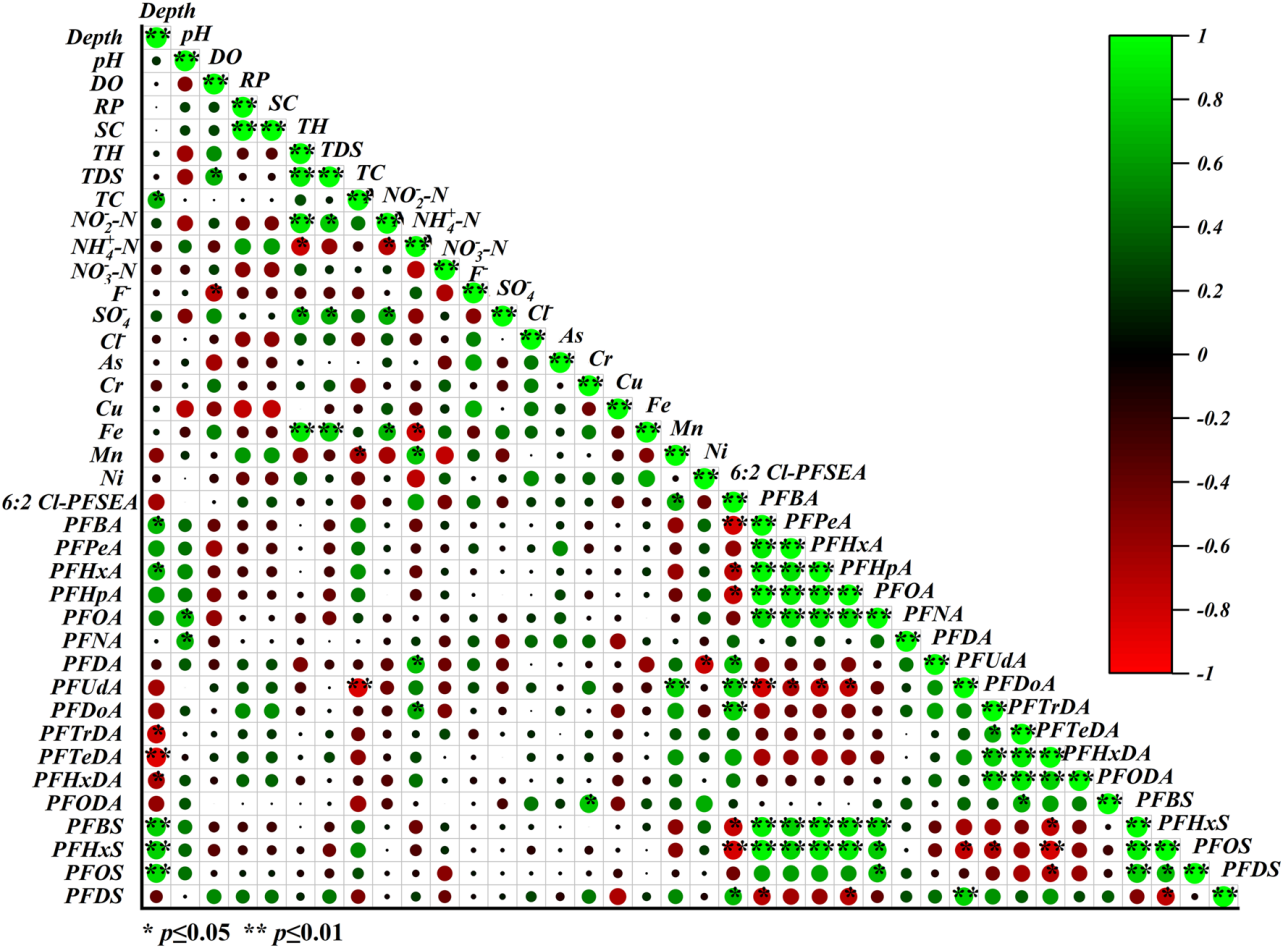
Correlations between physicochemical properties and multiple pollutants in groundwater from the FCP of Fuxin City, Liaoning Province.

Correlations among various pollutants and physicochemical properties (see Supplementary Table S6 online) are shown in Fig. 2. The relationship between F^−^ concentration and physicochemical properties was weak in groundwater except for DO, and the result was the same as that of Loganathan et al. for soil^41^. The F^−^ concentration had a strongly negative relationship (R^2^ = − 0.717, *p* = 0.02) with DO, which indicates that DO content may influence F^−^ migration or behaviour, but this also needs to be studied further. In the present study, the weak correlation between F^−^ and heavy metals was due to frequent rain, which dilutes the pollutants in water. The F^−^ in groundwater has many sources, including atmospheric deposition, agricultural activities, geographical factors, fertilizers and pesticides^42^. Although the F^−^ concentration had no significant relationship with TH (R^2^ = − 0.337, *p* = 0.3) in groundwater, F^−^ concentrations are continuously enriched, even after the groundwater reaches equilibrium with respect to CaF_2_ levels, due to removal of Ca by precipitation of calcite (CaCO_3_)^42,43^. In addition, plant cover type and amount, rain and soil properties also affect the migration of F^−^ from the surface to groundwater^40^. The F^−^ concentration showed the only significant relationship with heavy metal levels (*p* < 0.1) in soils from Shifang County of Sichuan Province; similarly, this relationship and the solubility of F^−^ in groundwater needs to be further studied^44^.

The environmental behaviour of PFASs in water bodies is influenced by various physicochemical properties, but the correlations between PFASs and physicochemical properties were limited in this study. pH, DO, RP, SC, TH, TDS and TC showed no significant positive or negative relationships with individual PFAS concentrations, whereas the water level had a positive correlation with short-chain PFASs and a negative correlation with long-chain PFASs. PFBA (R^2^ = 0.703, *p* = 0.03), PFBS (R^2^ = 0.803, *p* = 0.009), PFPeA (R^2^ = 0.619, *p* = 0.052), PFHxA (R^2^ = 0.710, *p* = 0.03), PFHxS (R^2^ = 0.802, *p* = 0.001), and PFHpA (R^2^ = 0.620, *p* = 0.054) had positive relationships with water level. PFASs tend to migrate to deep groundwater and soil along the path for movement and penetration of water. Short-chain PFASs were more inclined to migrate in water environments than long-chain PFASs because of their hydrophilicities. In addition, frequent rainfall may also accelerate flow migration and affect the PFAS concentration distribution in the groundwater system. Nevertheless, long chain PFDoA (R^2^ = − 0.607, *p* = 0.07), PFTrDA (R^2^ = 0.786, *p* = 0.01), PFTeDA(R^2^ = − 0.807, *p* = 0.002) and PFHxDA(R^2^ = − 0.670, *p* = 0.04) showed negative relationships with the water level. The strong hydrophobicities of long chain PFAS facilitate adsorption by particles in water and limit migration and mobility capabilities. The correlations indicate the fates of short- and long-chain PFASs in groundwater. The positive relationships between short chain PFASs or long chain PFASs indicates that carbon chain length plays a key role in migration. The solubility in water in the water body is inversely proportional to the carbon chain length^32^. A previous study from New Jersey, USA, also showed that long chain PFDA, PFUdA and PFDoA have higher affinities for organic material and preferentially partition into sediments^45^. Importantly, there were no relationships between PFASs and F^−^ or heavy metal concentrations in our study. The reason may be that PFASs and heavy metals come from different sources or their environmental concentrations are low, but this requires more study.

### Health risk assessments

**Figure 3:**
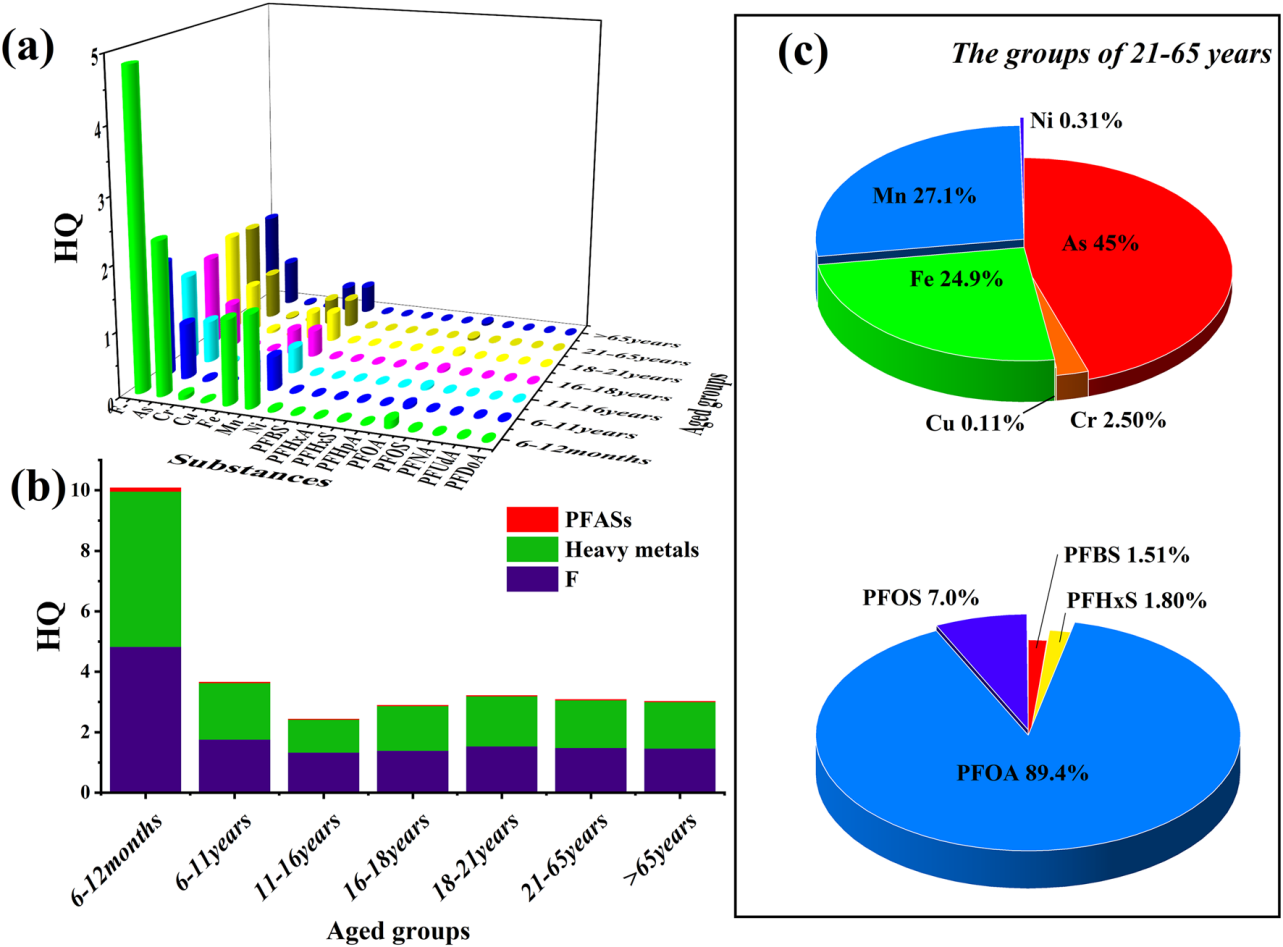
Health risk assessment for heavy metals, F^−^ and PFASs in groundwater from the FCP of Fuxin City, Liaoning Province; (**a**) HQ value for different age groups; (**b**) HQ_mix_ value for different age groups and (**c**) HQ contributions from different heavy metals and individual PFASs for subjects aged 21–65 year old.

Health risk assessments are widely used to evaluate harmful effects on the human body. The assessment method with the quotient is a simple model that effectively calculates the risk values for different pollutants in the groundwater system. However, the model only considers the risk of a single pollutant and does not apply complex mechanisms for different pollutants. The main detected pollutants, including heavy metals, individual PFASs, and F^−^, were assessed, and the results are shown in Fig. 3.

Figure 3a shows that children aged 6–12 months were more vulnerable to toxic pollutants than residents in the other age groups. The mean HQ values for heavy metals and F^−^ reached 4.82 and 5.13, respectively, which poses potential risk for the 6–12 month age group. The HQ value reflected a decreasing trend with increasing age. The mean HQ or HQ_mix_ value for F or heavy metals exceeded 1 for all age groups, which indicates that residents may suffer the negative effects of drinking contaminated groundwater. In Fig. 3b, the potential sources of risk for adults aged 21–65 years came from the heavy metals As (45.0%), Mn (27.1%), and Fe (24.9%). Although the quotients for As, Mn, and Fe were not exceeded 1 for the 21–65 year group, the HQ values were 2.34, 1.41, and 1.29 in the 6–12 month group (Fig. 3c). In studies of the risk due to heavy metals, groups of infants were exposed to toxic pollutants^46–49^. Abundant CaF_2_ resources were the main reason for the high F^−^ concentrations in groundwater, and earlier industrial production was also identified as another source. The HQ value for F^−^ exceeded 1 for all age groups (4.82 for 6–12 months, 1.75 for 6–11 years, 1.32 for 11–16 years, 1.38 for 16–18 years, 1.53 for 18–21 years, 1.47 for 21–65 years and 1.45 for > 65 years) (Fig. 3c). A previous study by Ozsvath et al. indicated that if a child ingests excess F^−^, a variety of adverse effects can occur in a healthy body, including dental fluorosis, skeletal fluorosis, increased rates of bone fractures and decreased birth rates^28^. Importantly, high F^−^ levels in groundwater are also introduced to farmland or residential areas by irrigation, although the risk from soil is lower than that from groundwater^19^. A study of a phosphate industrial area from Sichuan Province found that the main exposure pathway for pollutants was ingestion or particulate inhalation by groups of children and adults, respectively, while the highest risk value was less than 3–4 times that in our study^22^. F^−^ and PFASs were the predominant substances in the FCP, especially PFOA and PFOS, which were the common PFASs in the groundwater system. The toxicities of long-chain PFASs are much higher than those of short-chain PFASs, and they accumulate more easily in the human body^50,51^. In Fig. 3b, PFOA accounted for the largest proportion of risk among PFASs (89.4%), followed by PFOS (7.0%) and PFHxS (1.80%). PFASs in teenagers may influence the reproductive system and character development of residents living near a Chinese fluorine chemical plant, as shown previously^52^. Drinking water provides the major ingestion pathway for PFASs, and they have a long half-life in the human body.

## Conclusion

Contaminant concentrations and correlations between F^−^, heavy metal, and PFAS levels were studied, and health risk assessments for different age groups living around an abandoned FCP were performed. F^−^, Fe and Mn concentrations exceeded the recommended limits of the drinking water standard, and the Cd, Cr, Cu, Ni, Pb and Zn concentrations were considerable. PFBA, PFBS and PFHxA were the predominant substances, whereas the PFOA concentration was much higher than the risk control value from the USEPA. The F^−^ concentration showed weak correlations with various physicochemical properties, except for DO, and heavy metal concentrations. A strongly positive relationship existed between short chain PFASs and water depth, while long chain PFASs had a negative relationship with water depth. Carbon length plays an important role in the migration for PFASs. Health risk assessments for all age groups demonstrated that the risk according to source decreased in the order F^−^ > heavy metals > PFASs, and HQ values decreased for older age groups. As, Fe and Mn in the heavy metal group and PFOA in the PFASs groups were identified as the main sources of risk, so these substances should be listed as priority pollutants in the local region. Different pollutants in groundwater originate from various sources, so measures should be adopted to control pollutants at different levels. Residents should also reduce the frequency with which they drink groundwater by using external water to decrease health risks. Our study clarified the characteristics of various pollutants in groundwater and analysed potential correlations to provide a reference for local pollution prevention and control.

## Supplementary Information

Supplementary Information.
