# Supplementary material for: Multiple pollutants in groundwater near an abandoned Chinese fluorine chemical park: concentrations, correlations and health risk assessments
Source: Sci Rep. 2022 Mar 1;12:3370. doi: 10.1038/s41598-022-07201-8 (PMC8888542; doi:10.1038/s41598-022-07201-8)
Supplement: Supplementary file 1 — Supplementary Information. [file 41598_2022_7201_MOESM1_ESM.pdf]

SUPPLEMENTARY INFORMATION

Multiple pollutants in groundwater near an abandoned Chinese fluorine chemical park: concentrations, correlations and health risk assessments

Jiaxi Tang[1,2,*], Yongle Zhu[1], Biao Xiang[1], Yu Li[1], Ting Tan[1], Ying Xu[1], Mengxue Li[1]

[1] College of Environmental Science and Engineering, Liaoning Technical University, Fuxin 123000, China; E-Mails: tangjiaxi1986@163.com (J.X.T.); Zhu_lll@163.com (Y.L.Z.); xiangbiao23@yeah.net (B.X.); liyu13781588762@163.com (Y.L.); tan18241030570@163.com (T.T.); zb0595@163.com(Y.X.); 18341840938@163.com (M.X.L.);

[2] Liaoning Academy of Agricultural Sciences, Shenyang 110161, China

* Corresponding author E-Mails: tangjiaxi1986@163.com (J.X.T.);

Tel: +0418-5110118 Fax: +0418-5110118

The target PFAS analytes were purchased from Absolute Standards (Hamden, CT, USA), including 13 perfluoroalkyl carboxylates (PFCAs) of perfluorobutanoic acid (PFBA), perfluoropentanoic acid (PFPeA), perfluorohexanoic acid (PFHxA), perfluoroheptanoic acid (PFHpA), perfluorooctanoic acid (PFOA), perfluorononanoic acid (PFNA), perfluorodecanoic acid (PFDA), perfluoroundecanoic acid (PFUdA), perfluorododecanoic acid (PFDoA), perfluorotridecanoic acid (PFTrDA), perfluorotetradecanoic acid (PFTeDA), perfluorohexadecanoic acid (PFHxDA) and perfluorooctadecanoic acid (PFODA), 4 perfluoroalkyl sulfonates (PFSAs) of perfluorobutane sulfonate (PFBS), perfluorohexane sulfonate (PFHxS), PFOS and perfluorodecane sulfonate (PFDS).

Then the mixture of internal standards (IS) was purchased from Wellington Laboratories including $^{13}C_4$-PFBA, $^{13}C_3$-PFBS, $^{13}C_2$-PFHxA, $^{18}O_2$-PFHxS, $^{13}C_4$-PFOA, $^{13}C_4$-PFOS, $^{13}C_2$-PFDA, $^{13}C_5$-PFNA, $^{13}C_2$-PFUdA, $^{13}C_2$-PFDoA, and $^{13}C_2$-PFTrDA.

Table S1. Experiment methods of physicochemical properties, heavy metals and F^-

Items	Test method and standard	LODs	LOQs	Unit	Name and model of instrument
pH	GB/T 5750.4-2006(5.1)	—	—	—	pH-meter (PHS-3C)
Dissolved oxygen (DO)	HJ 506-2009	—	—	mg/L	Dissolved Oxygen Meter (JPBJ 608)
Redox potential(RP)	SL 94-1994	—	—	mV	pH-meter (PHS-3C)
Specific conductance(SC)	GB/T 5750.4-2006(6.1)	—	—	μS/cm	Conductivity meter (DDS-307A)
Total hardness(TH, $CaCO_3$)	GB/T 5750.4-2006 (7.1)	1.0	3.33	mg/L	Acid buret (50mL)
Total dissolved solids(TDS)	GB/T 5750.4-2006	—	—	mg/L	Shimadzu Corporation (ME204E02)
Total coliform (TC)	GB/T 5750.12-2006（2.1）	—	—	MPN/100mL	Biochemical incubator (SHP-250)
NO_2^--N	GB/T 5750.5-2006(10.1)	0.001	0.003	mg/L	UV-VIS Spectrophotometer
NO_3^--N	GB/T 5750.5-2006	0.02	0.07	mg/L	UV-VIS Spectrophotometer
NH_4^+-N	GB/T 5750.5-2006(5.3)	0.038	0.13	mg/L	Ion chromatograph (DIONEX-AQUION)
F^-	GB/T 5750.5-2006(3.2)	0.025	0.08	mg/L	Ion chromatograph (DIONEX-AQUION)
SO_4^-	GB/T 5750.5-2006(1.2)	0.188	0.63	mg/L	Ion chromatograph (DIONEX-AQUION)
Cl^-	GB/T 5750.5-2006(2.2)	0.038	0.13	mg/L	Ion chromatograph (DIONEX-AQUION)
As	GB/T 5750.6-2006(6.1)	-	-	mg/L	Atomic fluorescence spectrophotometer (PF32)
Cd	DZ/T 0064.80-1993	0.33	1.10	μg/L	Inductively coupled plasma source mass spectrometer (iCAP RQ)

Items	Test method and standard	LODs	LOQs	Unit	Name and model of instrument
Cr	GB/T 5750.6-2006(10.1)	-	-	mg/L	UV-VIS Spectrophotometer
Cu	DZ/T 0064.80-1993	0.33	1.10	μg/L	Inductively coupled plasma source mass spectrometer (iCAP RQ)
Fe	GB/T 5750.6-2006	0.1	0.33	mg/L	Atomic absorption spectrophotometer (A3AFG-12)
Hg	GB/T 5750.6-2006（8.1）	0.000025	0.00008	mg/L	Atomic fluorescence spectrophotometer (PF32)
Mn	DZ/T 0064.80-1993	0.33	1.10	μg/L	Inductively coupled plasma source mass spectrometer (iCAP RQ)
Ni	GB/T 5750.5-2006(3.2)	0.025	0.08	μg/L	Ion chromatograph (DIONEX-AQUION)
Pb	DZ/T 0064.80-1993	0.67	2.23	μg/L	Inductively coupled plasma source mass spectrometer (iCAP RQ)
Zn	DZ/T 0064.80-1993	0.33	1.10	μg/L	Inductively coupled plasma source mass spectrometer (iCAP RQ)

Table S2. The abbreviations of the studied PFASs

Classification	Abbreviation	Compound name	Carbon-chain length
PFCAs Perfluoroalkyl carboxylates	PFBA	Perfluorobutanoic acid	4
	PFPeA	Perfluoropentanoic acid	5
	PFHxA	Perfluorohexanoic acid	6
	PFHpA	Perfluoroheptanoic acid	7
	PFOA	Perfluorooctanoic acid	8
	PFNA	Perfluorononanoic acid	9
	PFDA	Perfluorodecanoic acid	10
	PFUdA	Perfluoroundecanoic acid	11
	PFDoA	Perfluorododecanoic acid	12
	PFTrDA	Perfluorotridecanoic acid	13
	PFTeDA	Perfluorotetradecanoic acid	14
	PFHxDA	perfluorohexadecanoic acid	16
	PFODA	Perfluorooctadecanoic acid	18
PFSAs Perfluoroalkyl sulfonates	PFBS	Perfluorobutane sulfonate	4
	PFHxS	Perfluorohexane sulfonate	6
	PFOS	Perfluorooctane sulfonate	8
	PFDS	Perfluorodecane sulfonate	10
Substitutes	6:2 Cl-PFSEA	Chlorinated polyfluorinated ether sulfonate acid	8

Table S3. Related paraments and information of HPLC–MS/MS

Number	Compounds	MS/MS transition (m/z)	Collision Energy (ms)	LODs (ng·L^{-1})	LOQs (ng·L^{-1})
1	PFBA	212.9→169.0<br>212.9→195.0	52	0.1	0.33
2	PFPeA	262.9→218.9<br>262.9→231.3	58	0.025	0.08
3	PFBS	298.9→80.1<br>298.9→99.0	144	0.025	0.08
4	PFHxA	312.9→91.1<br>312.9→268.8	75	0.025	0.08
5	PFHpA	362.9→168.9<br>362.9→318.9	73	0.025	0.08
6	PFHxS	398.9→80.1<br>398.9→99.1	204	0.01	0.03
7	PFOA	412.9→218.8<br>412.8→368.9	82	0.025	0.08
8	PFNA	462.9→218.9<br>462.9→418.9	86	0.025	0.08
9	PFOS	498.9→80.1<br>498.9→99.1	241	0.015	0.05
10	6:2 Cl-PFSEA	531.0→350.8<br>531.0→366.8	208	0.01	0.03
11	PFDA	512.9→268.8<br>512.9→468.8	105	0.01	0.03
12	PFUdA	562.9→268.9<br>562.9→518.9	106	0.01	0.03
13	PFDS	598.9→80.9<br>598.9→99.0	298	0.01	0.03
14	PFDoA	612.9→318.9<br>612.9→568.9	118	0.025	0.08
15	PFTrA	662.9→319.3<br>662.9→618.9	122	0.02	0.07
16	PFTeDA	712.9→418.9<br>712.9→668.9	133	0.03	0.10
17	PFHxDA	812.9→519.0<br>812.9→768.9	156	0.02	0.07
18	PFODA	912.9→419.3<br>912.9→868.8	166	0.02	0.07

Table S4. HPLC–MS/MS instrument conditions

Model	DIONEX UltiMate 3000 RS Autosampler<br>TSQ ENDURA
Column temperature	40 ℃
Injection volume	5 μL
Mobile phase	A= 10 mM ammonium acetate/methanol<br>B= 0.1mM ammonium acetate
Run time	15 min
Flow rate	0.3 mL/min
Positive Ion(V)	3500
Negative Ion(V)	3000
Sheath Gas (Arb)	50
Aux Gas(Arb)	10
Ion Transfer Tube Temp(℃)	350
Vaporizer Temp(℃)	400

Table S5. The parament values of health risk assessment

Parameters	Implication	Value		Unit
ADI	Acceptable Daily Intake	PFOA	1.5	μg/ kg day^{-1}
		As	0.3	
		Cr	3.0	
		Cu	40	
		Fe	300	
		Mn	140	
		Ni	20	
		F^-	0.04	
BW	Body weight	6-12 months	9.1	kg
		6-11 years	29.3	
		11-16 years	54.2	
		16-18years	67.6	
		18-21 years	67.6	
		21-65 years	78.8	
		>65 years	80.0	
DWI	Drinking Water Intake in daily life	6-12 months	1	L day^{-1}
		6-11 years	1.32	
		11-16 years	1.82	
		16-18years	1.78	
		18-21years	2.34	
		21-65 years	2.94	
		>65 years	2.73	
AB	Gastrointestinal absorption rate	Assumed to be equal to 1		-
FOE	Frequency of Exposure	0.96 (350/365 days)		-
C	PFASs concentration	From data		ng·L^{-1}

References: [8-17]

Table S6. The physicochemical properties in groundwater from FCP of Fuxin City

Indicators	Units	G1	G2	G3	G4	G5	G6	G7	G8	G9
pH	-	7.7	7.5	7.6	7.5	7.7	7.8	7.0	7.0	7.1
Dissolved oxygen (DO)	mg/L	4.62	2.83	1.92	4.33	4.07	1.63	5.08	4.44	3.62
Redox potential(RP)	mV	374	358	350	361	351	356	354	355	347
Specific conductance(SC)	μS/cm	374	358	350	361	351	356	354	355	347
Total hardness(TH, $CaCO_3$)	mg/L	699	470	240	701	791	611	887	901	911
Total dissolved solids(TDS)	mg/L	301	241	114	266	287	242	314	355	332
Total coliform (TC)	MPN/100mL	46	49	16	130	110	240	170	49	79
Depth	m	2.6	2	3.5	5.3	4.75	5.2	3.85	3.5	3.35

Reference

1. GB/T 5750.4-2006. Standard examination methods for drinking water-Organoleptic and physical parameters. National standards of the People's Republic of China, Beijing, China (2006).
2. HJ 506-2009. Water quality-Determination of dissolved oxygen-Electrochemical probe method. National standards of the People's Republic of China, Beijing, China (2009).
3. SL 94-1994. Determination of oxidation-reduction potential(Electromatric method). National standards of the People's Republic of China, Beijing, China (1994).
4. GB/T 5750.12-2006. Standard examination methods for drinking water-Microbiological parameters. National standards of the People's Republic of China, Beijing, China (2006).
5. GB/T 5750.5-2006. Standard examination methods for drinking water-Nonmetal parameters. National standards of the People's Republic of China, Beijing, China (2006).
6. GB/T 5750.6-2006. Standard examination methods for drinking water-Metal parameters. National standards of the People's Republic of China, Beijing, China (2006).
7. DZ/T 0064.80-1993. Standard examination methods for groundwater water quality. National standards of the People's Republic of China, Beijing, China (1993).
8. Cao, X. *et al.* Occurrence, sources and health risk of polyfluoroalkyl substances (PFASs) in soil, water and sediment from a drinking water source area. *Ecotoxicol Environ Saf* **174**, 208-217, doi:10.1016/j.ecoenv.2019.02.058 (2019).
9. Etchepare, R. & van der Hoek, J. P. Health risk assessment of organic micropollutants in greywater for potable reuse. *Water Res* **72**, 186-198, doi:10.1016/j.watres.2014.10.048 (2015).
10. Ojemaye, C. Y. & Petrik, L. Occurrences, levels and risk assessment studies of emerging pollutants (pharmaceuticals, perfluoroalkyl and endocrine disrupting compounds) in fish samples from Kalk Bay harbour, South Africa. *Environ Pollut* **252**, 562-572, doi:10.1016/j.envpol.2019.05.091 (2019).
11. Qi, Y. *et al.* Identification, characterization, and human health risk assessment of perfluorinated compounds in groundwater from a suburb of Tianjin, China. *Environmental Earth Sciences* **75**, doi:10.1007/s12665-016-5415-x (2016).
12. Sun, R. *et al.* Perfluorinated compounds in surface waters of Shanghai, China: Source analysis and risk assessment. *Ecotoxicol Environ Saf* **149**, 88-95, doi:10.1016/j.ecoenv.2017.11.012 (2018).
13. Wang, Y., Shi, Y. & Cai, Y. Spatial distribution, seasonal variation and risks of legacy and emerging per- and polyfluoroalkyl substances in urban surface water in Beijing, China. *Sci Total Environ* **673**, 177-183, doi:10.1016/j.scitotenv.2019.04.067 (2019).
14. Chen, L. *et al.* Heavy metals in food crops, soil, and water in the Lihe River Watershed of the Taihu Region and their potential health risks when ingested. *Sci Total Environ* **615**, 141-149, doi:10.1016/j.scitotenv.2017.09.230 (2018).
15. Khan, S., Cao, Q., Zheng, Y. M., Huang, Y. Z. & Zhu, Y. G. Health risks of heavy metals in contaminated soils and food crops irrigated with wastewater in Beijing, China. *Environmental Pollution* **152**, 686-692, doi:10.1016/j.envpol.2007.06.056 (2008).
16. Tang, J., He, M., Luo, Q., Adeel, M. & Jiao, F. Heavy Metals in Agricultural Soils from a Typical Mining City in China: Spatial Distribution, SourceApportionment, and Health Risk Assessment. *Polish Journal of Environmental Studies* **29**, 1379-1390, doi:10.15244/pjoes/108517 (2020).
17. Zhang, C., Shan, B., Tang, W., Wang, C. & Zhang, L. Identifying sediment-associated toxicity in rivers affected by multiple pollutants from the contaminant bioavailability. *Ecotoxicol Environ Saf* **171**, 84-91, doi:10.1016/j.ecoenv.2018.12.075 (2019).
